# Influence of introduced vs. native parasites on the body condition of migrant silver eels

**DOI:** 10.1051/parasite/2013040

**Published:** 2013-10-21

**Authors:** Claudia Gérard, Thomas Trancart, Elsa Amilhat, Elisabeth Faliex, Laure Virag, Eric Feunteun, Anthony Acou

**Affiliations:** 1 ECOBIO, CNRS, Université de Rennes 1 avenue du Général Leclerc 35042 Rennes France; 2 UMR 7208 BOREA, CRESCO, Muséum National d’Histoire Naturelle 38 rue du Port Blanc 35800 Dinard France; 3 CNRS, Centre de Formation et de Recherche sur les Environnements Méditerranéens, UMR 5110 66860 Perpignan France; 4 Université de Perpignan Via Domitia, Centre de Formation et de Recherche sur les Environnements Méditerranéens, UMR 5110 66860 Perpignan France

**Keywords:** *Anguilla anguilla*, Silver eels, Metazoan parasite communities, Introduced parasites, Body condition

## Abstract

Because parasitism is among the reasons invoked to explain the collapse of *Anguilla anguilla*, we evaluated the parasitic constraint on body condition (BC) of migrant silver eels as a proxy of fitness with inter-site comparisons. Metazoan parasites were studied in 149 silver eels from five sites (northern Europe). In total, 89% were infected by 13 species including Myxozoa, Monogenea, Cestoda, Nematoda, and Acanthocephala. *Anguillicoloides crassus* was most common (56%), then *Acanthocephalus clavula* (30%), and *Pseudodactylogyrus* sp. (17%). BC, calculated for 58 females, was negatively correlated by abundance of the introduced *Pseudodactylogyrus* sp. but not by other parasite taxa. Nevertheless, the introduced *A. crassus* was considered as a severe pathogen based on previous data, whereas the native *A. clavula* was supposed to have limited impact. Parasite component communities and BC were different between sites. Silver eels from Stockholm Archipelago (Sweden) were the least parasitized (40% vs. 90–95% for other sites) with no parasites on the gills. Burrishoole (Ireland) differed by the absence of *A. crassus* and high prevalence of *A. clavula* (84%) but without consequences on BC. Gudenaa (Denmark), Corrib (Ireland), and Frémur (France) were close due to high prevalence of *A. crassus* (89–93%). Gudenaa and Corrib were the most similar because *Pseudodactylogyrus* sp. was also highly prevalent (respectively 71% and 60%) whereas absent in Frémur. Our results suggest that the fitness loss induced by the introduced parasites could affect the spawning success of migrant silver eels from Gudenaa and Corrib, and to a lesser extent from Frémur, but probably not those from Stockholm Archipelago and Burrishoole.

## Abstract (fr)

Parce que le parasitisme figure parmi les raisons évoquées pour expliquer le déclin d’*Anguilla anguilla*, nous avons évalué la contrainte parasitaire sur l’indice de condition (BC) comme mesure de la fitness chez des anguilles argentées en cours de migration avec une comparaison entre sites. Les métazoaires parasites ont été étudiés chez 149 anguilles argentées de 5 sites (Europe du Nord). Au total, 89 % étaient infectées par 13 espèces parmi les Myxozoa, Monogenea, Cestoda, Nematoda et Acanthocephala. *Anguillicoloides crassus* était la plus commune (56 %), puis *Acanthocephalus clavula* (30 %), et *Pseudodactylogyrus* sp. (17 %). Le BC, calculé pour 58 femelles, était négativement influencé par l’abondance du parasite introduit *Pseudodactylogyrus* sp. mais pas par les autres taxons. Néanmoins, *A. crassus* est considérée comme un pathogène introduit sévère selon les données déjà publiées, alors que l’espèce native *A. clavula* est supposée avoir un impact limité. Les communautés parasitaires et le BC étaient différents selon les sites. Les anguilles argentées de Stockholm Archipelago (Suède) étaient les moins parasitées (40 % vs. 90–95 % pour les autres sites) et n’abritaient pas de parasites dans leurs branchies. Burrishoole (Irlande) différait par l’absence d’*A. crassus* et une forte prévalence d’*A. clavula* (84 %) mais sans conséquences sur le BC. Gudenaa (Danemark), Corrib (Irlande) et Frémur (France) étaient proches en raison de la prévalence élevée d’*A. crassus* (89–93 %). Gudenaa et Corrib étaient les plus similaires car *Pseudodactylogyrus* sp. était aussi fortement prévalent (respectivement 71 % et 60 %) mais absent dans le Frémur. Nos résultats suggèrent que la perte de fitness induite par les parasites introduits pourrait affecter le succès de la reproduction des anguilles argentées migrantes originaires de Gudenaa et Corrib, et dans une moindre mesure du Frémur, mais probablement pas de celles de Stockholm Archipelago et Burrishoole.

## Introduction

In spite of the perennial scientific mysteries about its biology and population genetic structure, the European eel *Anguilla anguilla* is considered as panmictic, therefore a breeder emigrating from a given river theoretically contributes to subsequent glass eel recruitment along the whole continental distribution range [5, 16, 21]. Depending on subpopulations and habitat characteristics, eels spend about 3–30 years in fresh and brackish waters of Europe and North Africa, growing and accumulating fat reserves for an active swimming migration as silver eel stage across the Atlantic Ocean and reproduction once in the Sargasso Sea [36]. The probability that silver eels reach the spawning grounds and reproduce successfully is likely to vary greatly among continental growing sites [4, 18]. A pan-European methodology to estimate the overall breeding potential of silver eels according to relevant criteria (e.g., fat composition, contamination by chemicals, parasitic load) is not ready for use because of the complexity in implementing such an approach. The use of silver eel condition indices may constitute a first step in this direction. Body condition has been demonstrated to indicate energy reserves of *Salmo salar* [59], *Coregonus artedi* [54], and *Gadus morhua* [25, 40]. Considering the importance of energy reserves during both transoceanic migration and reproduction of the European eel [7], we hypothesize that body condition may represent a good proxy of silver eel fitness [4, 31].

Because most parasitized organisms are generally in poorer condition than unparasitized ones [15, 22, 61 for reviews] and because parasites interact with natural and anthropogenic stressors to increase mortality and reduce animal health in myriad ways [47 for review], parasitism is among the reasons invoked to explain the European eel collapse [9, 20, 28, 35]. Up to now, 161 species or taxa have been described in *A. anguilla* from fresh, brackish, and marine waters in 30 countries of Europe and North Africa [32 for review]. Among them, the introduced *Anguillicoloides crassus* (swimbladder nematode) and *Pseudodactylogyrus* sp. (gill monogenean) are considered as an important factor able to induce a stress that probably decreases the host fitness and seriously hampers the recovery of the European eel [for reviews: 9, 35]. The scientific research program EELIAD (2008–2012) aimed to resolve some of the mysteries of eel biology in order to help conserving European eel stocks. EELIAD provided us the opportunity to investigate the parasitism patterns of some organs (gills, heart, intestine, and swimbladder) of silver eels sampled at the start of their transoceanic migration across five far-distant sites from northern Europe, i.e., Sweden, Denmark, Ireland (two sites), and France. Our objectives were (i) to describe the metazoan parasite community of these five silver eel subpopulations, and (ii) to evaluate in each site the potential parasitic constraint (on the whole and depending on parasite taxa) on the body condition of silver eels. Because a greater pathogenicity is often observed for recent (vs. long-term coevolved) host-parasite associations as for *Pseudodactylogyrus* sp. and *A. crassus* in *A. anguilla* [33, 34, 48], we suppose that the least parasitized silver eels by these introduced species could be the most susceptible to reach the spawning grounds and to reproduce in the Sargasso Sea compared to the most heavily infected ones.

## Materials and methods

### Study areas and silver eel sampling (Figure 1; Table 1)

Silver eels (149) were caught during their seaward migration between October and February (i.e., at the beginning of their transoceanic migration) in five coastal water bodies from northern Europe: Stockholm Archipelago in Sweden (SWE-ARCH, *n* = 10, Oct 2009), River Gudenaa in Denmark (DEN-GUD, *n* = 21, Dec 2009), Burrishoole (IRE-BUR, *n* = 49, Nov 2008), and Corrib (IRE-COR, *n* = 26, Nov 2009) in Ireland, and River Frémur in France (FRA-FRE, *n* = 43, Feb 2010). The eel collection was focused on silver eel stage at the same maturation degree (see below the three criteria for silver eel assessment). Migrant silver eels were intercepted using pound nets (SWE-ARCH), coghill nets (IRE-COR), or eel-traps installed on dams (DEN-GUD, IRE-BUR, and FRA-FRE) depending on the site, these different methods of capture being adapted to both the size of the eels and the studied systems. Water bodies sampled differed in trophic status and level of anthropogenic pressure (Table 1). Among sites, IRE-BUR is considered as the pristine reference site due to the clean water and null anthropogenic pressure (no fishery, controls on movement of boat, water, or fishing gear).

**Figure 1.**
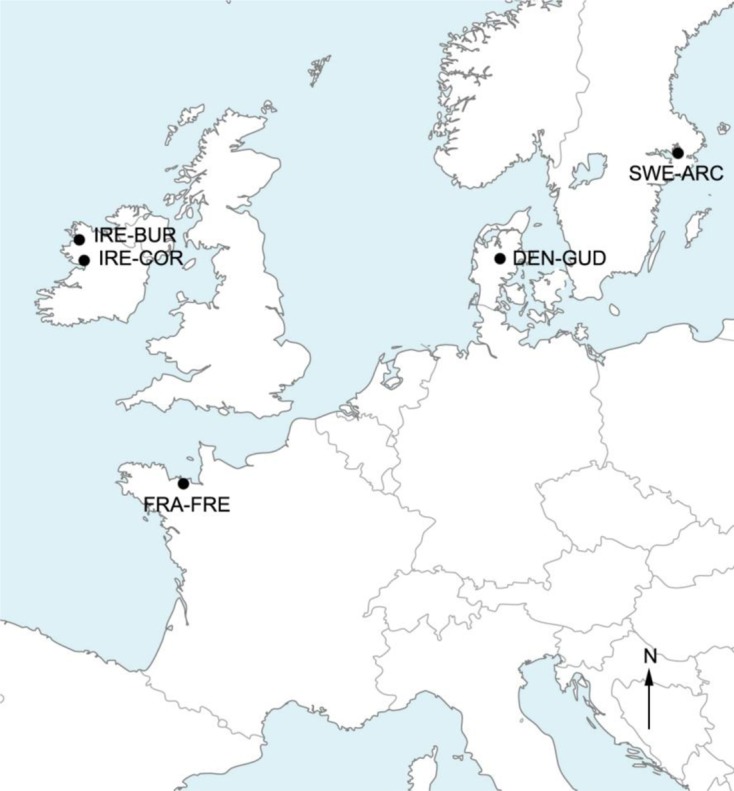
Geographical position of the sample sites of *Anguilla anguilla* in northern Europe: Burrishoole (IRE-BUR) and Corrib (IRE-COR) in Ireland, Frémur in France (FRA-FRE), Gudenaa in Denmark (DEN-GUD), and Stockholm Archipelago in Sweden (SWE-ARC).

**Table 1.** Characteristics of the five coastal water bodies sampled, i.e., Stockholm Archipelago (SWE-ARC), Gudenaa (DEN-GUD), Burrishoole (IRE-BUR), Corrib (IRE-COR), and Frémur (FRA-FRE). Trophic status according to Carlson (1977) [12]. Sampling dates from October 2009 to February 2010 correspond to the same seaward migration of the silver eels whereas sampling in Burrishoole occurred one-year sooner.

Country	Sampling site	Latitude (N)	Longitude (W or E)	Number of silver eels	Sampling date	Salinity (psu)	Distance from the sea (km)	Trophic status	Anthropogenic pressure
Sweden	SWE-ARC	58°57′30.82″	18°02′05.09″ E	10	Oct-2009	5	0	Mesotrophic	Low
Denmark	DEN-GUD	55°58′01.31″	09°42′16.64″ E	21	Dec-2009	0	100	Mesotrophic	Moderate
Ireland	IRE-BUR	53°55′13.51″	09°35′03.30″ W	49	Nov-2008	0	5.0	Oligotrophic	Null
Ireland	IRE-COR	53°16′32.05″	09°03′21.71″ W	26	Nov-2009	0	0.6	Oligotrophic	Low
France	FRA-FRE	48°34′39.80″	02°06′13.10″ W	43	Feb-2010	0	4.5	Eutrophic	High

### Silver eels: Development stage and sex determination, aging by otoliths

Each fish collected was anesthetized with Benzocaine at a concentration of 0.15 g L^−1^. Then, total weight (TW, g) and total length (TL, mm) were measured respectively to the nearest g and mm. Silver eels were identified by three criteria [1, 2]: color of the back and belly, presence of a well-defined lateral line, as well as ocular index (*OI* ≥ 6.5, Pankhurst’s 1982).

The sex was assigned by macroscopic observation of the gonads using the criteria described by Colombo et al. [14]. Because most silver eels sampled were females (132 vs. 17 males), we only selected female eels to study the parasitic impact on the eel body condition.

As a proxy of fitness, we used index developed by Le Cren [41] by calculating the body condition (BC) as the relative total weight TWr = 100 TW/TWstd, where TWstd is the predicted standard total weight of a fish at the same TL, as calculated according to the log_10_TW − log_10_TL regression equation (least squares means fit) for the whole sample of 132 female silver eels.

A sample of 58 female silver eels (SWE-ARCH, *n* = 4; DEN-GUD, *n* = 10; IRE-BUR, *n* = 17; IRE-COR, *n* = 11; FRA-FRE, *n* = 16) was aged by examination of the sagittal otoliths. The extracted otoliths were glued dorsal side up with crystal bond, grounded along the longitudinal plane until the nucleus was reached. Following the method described by the EIFAC ICES working group on eel [30], the fish continental age (number of years in fresh water) was determined through a stereomicroscope by counting the number of annuli from the first growth check outside the so-called zero band. This band is commonly assumed as the beginning of eel’s continental growth.

### Parasitological research in sampled *Anguilla anguilla*

All the 149 silver eels sampled were frozen before the research of metazoan parasites made on the gills, the heart, the digestive tract, and the swimbladder (the other organs were not available for parasitic investigation). Each organ was dissected using binocular microscope and all the metazoan parasites were numbered per organ and per eel (except for Myxozoa for which the presence or absence of cysts was recorded), identified and preserved in alcohol 70°. The parasitological parameter used to describe the community structure of parasites was species richness (number of parasite species in a sample of hosts); those to describe parasite populations (for a given parasite species) were prevalence (number of hosts infected with a particular parasite species/number of examined hosts), mean abundance (average abundance of a parasite species among all members of a host sample), and mean intensity (total number of parasites of a particular species found in a sample divided by the number of hosts infected with that parasite) [10].

### Statistical analyses

All the statistical analyses were made with the R-Cran project free software (http://www.r-project.org/). Differences were considered statistically significant at *p* < 0.05. Mean values of data are reported as means ± standard error (*SE*) except for prevalences ± 95% confidence limits (*CL*).

#### Inter-site comparison of the communities of parasites

Taxa with low prevalence (≤ 2%) were excluded of the analysis (*Acanthocephalus lucii*, *Echinorhynchus truttae*, *Eustrongylides* sp., *Pomphorhynchus laevis*, and *Raphidascaris acus*) because of their rarity and probable limited impact on silver eel subpopulations.

**Figure 2.**
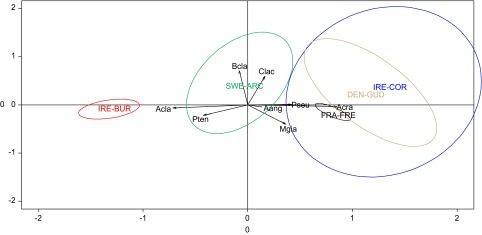
Factorial map according to the Multiple Correspondence Analysis on the presence/absence of the eight parasite taxa [*Acanthocephalus anguillae* (Aang), *Acanthocephalus clavula* (Acla), *Anguillicoloides crassus* (Acra), *Bothriocephalus claviceps* (Bcla), *Camallanus lacustris* (Clac), *Myxidium giardia* (Mgia), *Paraquimperia tenerrima* (Pten), and *Pseudodactylogyrus* sp. (Pseu)] in silver eels from the five study-sites (DEN-GUD, FRA-FRE, IRE-BUR, IRE-COR, and SWE-ARC).

**Figure 3.**
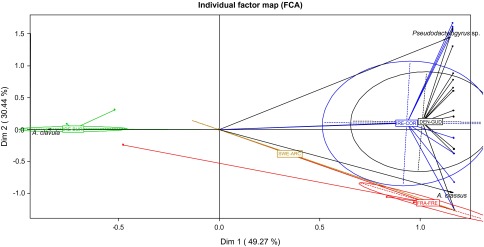
Factorial map according to the Multiple Correspondence Analysis on the abundance of the three major parasite taxa (*Acanthocephalus clavula*, *Anguillicoloides crassus*, and *Pseudodactylogyrus* sp.) in silver eels from the five study-sites (DEN-GUD, FRA-FRE, IRE-BUR, IRE-COR, and SWE-ARC)

First, the structure of the component parasite communities in silver eels (*n* = 149) among the five sites was detected and represented by a Multiple Correspondence Analysis (MCA, Ade4 package) performed on the presence/absence of eight parasite taxa (*Myxidium giardi*, *Pseudodactylogyrus* sp., *Bothriocephalus claviceps*, *A. crassus*, *Paraquimperia tenerrima*, *Camallanus lacustris*, *Acanthocephalus clavula*, and *Acanthocephalus anguillae*). Second, similar tests (MCA) were focused on the abundances of the three major parasite taxa (i.e., the two introduced *A. crassus* and *Pseudodactylogyrus* sp., and the native *A. clavula*) that could exert a significant constraint on silver eels, potentially compromising the migration and spawning success [28, 35]. In factorial maps (Figures 2 and 3) realized from MCA results, ellipses are only visual summary. The probability to be located in the ellipse is *p* = 1 − exp(−k^2^/2) with *k* = 1.5 showing that around 67% of observations were located in the ellipses.

#### Factors influencing the body condition of female silver eels

Five variables which could explain BC were chosen: the age of female silver eels, the study-site, and the number of each of the three major parasite taxa (*A. crassus*, *A. clavula*, and *Pseudodactylogyrus* sp.). Their influence was analyzed using generalized linear models (glm, base package). We tested all possible combinations with these variables including interactions. Nineteen different models were tested and compared using classical selection by both step-akaike information criterion corrected from small sample sizes (AICc) and deviance explained (DE) [29]. Then, the effect of parameters was analyzed by analysis of variance (ANOVA) from the selected model. As the sample size (58) used for the glm analyses was small according to the number of study-sites and variables tested, a power test using pwr package (pwr.f2.test) was performed on the selected model in order to give confidence in the acceptation of the null hypothesis (the fitness loss was not affected in some localities).

## Results

### Composition of the metazoan parasite community

A total of 149 European silver eels were dissected among them 89 ± 5% (132) were infected by one to six metazoan parasite taxa among the 13 identified in the whole sampling (Table 2). No trematodes were found. The nematode *A. crassus*, the only species recorded in the swimbladder, was the most common (prevalence of 56 ± 8%, mean intensity of 9.01 ± 1.19, and mean abundance of 4.98 ± 0.76), followed by *A. clavula* (30 ± 7%, 28.16 ± 4.17, and 8.45 ± 1.64) in the intestine, and then *Pseudodactylogyrus* sp. (17 ± 6%, 18.78 ± 4.73, and 2.47 ± 0.78) on the gills.

**Table 2.** Metazoan parasite species and their ecological parameters in *Anguilla anguilla* (149) from the five study-sites (CL 95% was calculated for each prevalence).

Species	Abbreviations	Prevalence %	CL95%	Microhabitat in eels	Diet
Myxozoa					
*Myxidium giardi* (Cépède, 1906) [13] (cysts)	Mgia	4.5	3.3	Gill	–
Monogena					
*Pseudodactylogyrus* sp.	Pseu	17.3	6.1	Gill	Surface browser
Cestoda					
*Bothriocephalus claviceps* (Goeze, 1782) [24]	Bcla	2.7	2.6	Intestine	Osmotrophe
Nematoda					
*Anguillicoloides crassus* (Kuwahara, Niimi & Hagaki, 1974) [39]	Acra	56.0	8.0	Swimbladder	Hematophagous
*Camallanus lacustris* (Zoega, 1776) [50]	Clac	2.7	2.6	Intestine	Hematophagous
*Paraquimperia tenerrima* (Linstow, 1878) [45]	Pten	4.7	3.4	Intestine	Chyle feeder
*Raphidascaris acus* (Bloch, 1779) [6]	Racu	1.3	1.8	Intestine	Chyle feeder
*Eustrongylides* sp. (cysts)	Eust	0.7	1.3	Stomach wall	–
Acanthocephala					
*Acanthocephalus anguillae* (Müller, 1780) [51]	Aang	6.0	3.8	Intestine	Osmotrophe
*Acanthocephalus lucii* (Müller, 1776) [50]	Aluc	2.0	2.3	Intestine	Osmotrophe
*Acanthocephalus clavula* Dujardin, 1845 [17]	Acla	30.2	7.4	Intestine	Osmotrophe
*Echinorhynchus truttae* Schrank, 1788 [57]	Etru	0.7	1.3	Intestine	Osmotrophe
*Pomphorhynchus laevis* (Müller, 1776) [50]	Plae	1.3	1.8	Intestine	Osmotrophe

### Component communities of metazoan parasites in each subpopulation of *Anguilla anguilla*

According to the MCAs (Figures 2 and 3), the structure of the parasite component communities (Table 3) varied among the study-sites both on the presence/absence of the total parasite taxa and on the abundance of the three most prevalent taxa (*A. crassus*, *A. clavula*, and *Pseudodactylogyrus* sp.).

**Table 3.** Component communities of metazoan parasites in *Anguilla anguilla* (149) from the five sampling sites: species richness, prevalence (*P*%), mean intensity (*I* ± *SE*) and mean abundance (*A* ± *SE*). See abbreviations in Tables 1 and 2.

	SWE-ARC (*N* = 10)			DEN-GUD (*N* = 21)			IRE-BUR (*N* = 49)			IRE-COR (*N* = 26)			FRA-FRE (*N* = 43)		
	*P*%	*I* ± *SE*	*A* ± *SE*	*P*%	*I* ± *SE*	*A* ± *SE*	*P*%	*I* ± *SE*	*A* ± *SE*	*P*%	*I* ± *SE*	*A* ± *SE*	*P*%	*I* ± *SE*	*A* ± *SE*
Mgia				4.8	–	–				20.0	–	–	7.0	–	–
Pseu				71.4	18.75 ± 5.29	14.29 ± 4.38	4.1	2.00 ± 1.00	0.08 ± 0.06	60.0	23.00 ± 11.59	13.80 ± 4.76			
Bcla	9.1	1.00	0.10 ± 0.10	9.5	1.50 ± 0.50	0.14 ± 0.10	2.0	1.00	0.02 ± 0.02	3.9	1.00	0.04 ± 0.04			
Acra	30.0	1.00 ± 0.00	0.30 ± 0.15	92.3	9.54 ± 1.50	8.27 ± 1.56				88.5	12.22 ± 3.38	10.81 ± 3.08	93.0	7.60 ± 1.20	7.07 ± 1.16
Clac	9.1	4.00	0.40 ± 0.40							7.7	22.50 ± 11.50	1.73 ± 1.36	2.3	1.00	0.02 ± 0.02
Pten							12.2	1.14 ± 0.14	0.16 ± 0.06						
Racu				9.5	1.50 ± 0.50	0.14 ± 0.10									
Eust				4.8	2.00	0.10 ± 0.10									
Aang				28.6	9.17 ± 7.94	3.53 ± 3.25				11.5	2.00 ± 0.58	0.23 ± 0.14			
Aluc										11.5	1.33 ± 0.33	0.15 ± 0.09			
Acla							83.7	30.51 ± 4.40	25.53 ± 4.02				9.3	5.00 ± 4.00	0.34 ± 0.30
Etru				4.8	1.00	0.14 ± 0.10									
Plae	9.1	3.00	0.30 ± 0.30				2.0	4	0.08 ± 0.08	3.9	2.00	0.08 ± 0.08			
Species Richness	4			8			5			8			4		
Total P% (CL)	40.0 (12/74)			95.2 (79/100)			89.8 (78/97)			92.3 (77/96)			93.0 (82/98)		

*Myxidium giardii* cysts (Mgia) were not numbered in the gills. CL95% was indicated in parentheses for total prevalence per site.

IRE-BUR strongly differed from the four other sites. First, it was the only site where *A. crassus* was completely absent. Second, the intestine was heavily infected with a total prevalence of 90% (*CL* = 78–97%), mostly by the acanthocephalan *A. clavula* that was highly occurrent in terms of prevalence (84% with *CL* = 71–93%), mean intensity (30.51 ± 4.40), and mean abundance (25.53 ± 4.02). A second site, SWE-ARC, was also significantly different from the others (even with the small sample size of 10 fish compared to other sites) due to an overall prevalence less than half (40% with CL = 12–74% vs. 90–95% with CL = 77–100% in other sites) and to a total absence of parasites on the gills (Table 3). The three other sites: DEN-GUD, IRE-COR, and FRA-FRE, were characterized by high prevalences of *A. crassus* varying from 89% (*CL* = 67–104%) to 93% (80–99%). Among all sites, DEN-GUD and IRE-COR were the most similar because of the highest species richness (eight parasite taxa vs. four and five in other sites) and of the highest prevalences of *Pseudodactylogyrus* sp.; no monogenean was found in silver eels from FRA-FRE (Table 3).

### Influence of parasitism on the body condition of female silver eels

Results from the 19 linear models are summarized in Table 4. According to the two criterions of selection (AICc and DE), the model #18 was selected. Results of ANOVA performed on this model are summarized in Table 5. The selected model explained 42.43% of the total deviance, and 33.10% of this explained deviance was explained only by the site (or 14.04% of total deviance, *p* = 0.013). The combined effect of female age and study-site was responsible of 27.70% of the explained deviance (*p* = 0.061). Among the three major parasite taxa, the number of *Pseudodactylogyrus* sp. had a strong negative effect on the BC of female silver eels (*p* = 0.003) and explained 27.10% of the explained deviance. The number of *A. crassus* tends to have a positive effect on the BC (*p* = 0.048). Model selection using step-AICc process showed that *A. clavula* had no significant effect on the BC. Indeed, AICc and explained deviance were not different between models #18 and #19, this latter being similar to #18 but with *A. clavula* as additional variable (Table 5).

**Table 4.** List of all the 18 models tested with AICc (akaike information criterion corrected from small sample sizes) and DE (deviance explained, %); BC = body condition.

No.	Model	AIC	DE
1	BC ~ site	393.13	14.04
2	BC ~ age	390.25	12.23
3	BC ~ *Pseudodactylogyrus* sp.	394.03	5.86
4	BC ~ *A. clavula*	396.10	2.19
5	BC ~ *A. crassus*	397.04	0.47
6	BC ~ site + age	389.05	23.19
7	BC ~ site + age + *Pseudodactylogyrus* sp.	384.96	31.38
8	BC ~ site + age:site + *Pseudodactylogyrus* sp. + *A. clavula*	387.90	37.51
9	BC ~ site + age + *Pseudodactylogyrus* sp. + *A. clavula* + *A. crassus*	384.83	36.42
10	BC ~ site + age + *Pseudodactylogyrus* sp.:site + *A. clavula*:site + *A. crassus*:site	389.27	40.49
11	BC ~ site + age:site + *Pseudodactylogyrus* sp.:site + *A. clavula*:site + *A. crassus*:site	389.27	46.74
12	BC ~ age:site + *Pseudodactylogyrus* sp.:site + *A. clavula*:site + *A. crassus*:site	388.67	41.14
13	BC ~ site + site:age + *Pseudodactylogyrus* sp.:site + *A. crassus*:site	385.78	46.24
14	BC ~ site + age:site + *Pseudodactylogyrus* sp.:site	387.90	37.51
15	BC ~ site + age:site + *Pseudodactylogyrus* sp.:site + *A. clavula*:site	391.39	38.10
16	BC ~ site + age + *Pseudodactylogyrus* sp.:site + *A. clavula*:site	390.44	32.04
17	BC ~ site + age + *Pseudodactylogyrus* sp. + *A. crassus*	382.84	36.42
18	BC ~ site + age:site + *Pseudodactylogyrus* sp. + *A. crassus*	382.44	42.43
19	BC ~ site + age:site + *Pseudodactylogyrus* sp. + *A. crassus* + *A. clavula*	385.47	42.43

**Table 5.** Deviance explained (%) by each significant variable (study-site, *Pseudodactylogyrus* sp., *Anguillicoloides crassus* and site:age) of the selected model.

Variables	Deviance	*P*-value
Study-site	14.04	0.013
*Pseudodactylogyrus* sp.	11.50	0.003
*A. crassus*	5.12	0.048
Site:age	11.77	0.061
Total deviance explained	42.43	0.002

## Discussion

Whatever the host-parasite combination, parasites and their host compete for resources in such a way that both survival and fecundity of the host could be affected, even if no pathology is obvious and even if this effect may be drowned in the background noise of all other factors that affect survival and fecundity [15, 22, 52, 61 for reviews]. Other environmental stressors (e.g., pollutants, pathogens) that can also influence the health status of the eels [3, 23, 26, 56] could be considered as a potential confounding factor, but in most cases, contaminants and parasites occurring together are shown to exacerbate the detrimental effects on individuals (synergistic effects), suggesting that parasitized fish in polluted environments are in a poorer condition than unparasitized fish [47 for review]. Thus, to consider parasitism and its impact can help to understand the collapse of the European eel. In our study, 89 ± 5% of all silver eels were parasitized and 13 metazoan taxa were identified including Myxozoa, Monogenea, Cestoda, Nematoda, and Acanthocephala commonly recorded in *A. anguilla* [32 for review]. No Trematoda were found from any site in spite of 39 trematode species described in *A. anguilla* as definitive host [32]. One possible explanation is that our silver eels were originated from waters with salinity ≤ 5 psu and that trematodes infecting eels are shown to be significantly less frequent in fresh vs. marine waters [37, 38].

The body condition of silver eels was used as a proxy of fitness that can reveal the impact of parasite infections. Among the three major parasite taxa recorded in the female silver eels studied here (*A. crassus*, *A. clavula*, and *Pseudodactylogyrus* sp.), only *Pseudodactylogyrus* sp. decreased the host body condition in relation with increasing abundance (up to 80 worms) on the gills. *Pseudodactylogyrus* sp. is the third more prevalent taxon in this study (17 ± 6%) and a specific gill monogenean of the genus *Anguilla* transferred from its native host *Anguilla japonica* to *A. anguilla* after introduction in Europe in 1977 [33, 35, 62]. *Pseudodactylogyrus* sp. is browsing the host gill surface and induces epithelial lesions potentially leading to lethal hypoxia of the highly susceptible European eel and facilitating infections by various opportunist pathogens (virus, bacteria, and fungi) [33, 35, 62].

In contrast with *Pseudodactylogyrus* sp., the infection by the introduced hematophagous *A. crassus*, the most prevalent species (56 ± 8%), had no significant effect on the condition factor of *A. anguilla*, as also shown by other studies [58 for review], and even tended to have a positive effect with increasing nematode abundance in the swimbladder. But this criterion is not the best reflecting the eel pathogenicity due to the short life cycle of *A. crassus*, and severely damaged swimbladders are shown to harbor very few or even no living nematodes [42, 63]. Nevertheless, using the Swimbladder Degenerative Index, Lefebvre et al. [44] recently demonstrated that the most affected eels had greater body length and mass (+11% and +41% respectively) than unaffected eels of the same age. Despite these surprising counterintuitive results, high virulence and severe impacts of *A. crassus* are expected because *A. anguilla* lacks an adaptive immune response, and various pathogenic potentially lethal effects (e.g., anemia, energy drain, swimming performance decrease) have been demonstrated, threatening the success of spawning migration in the Sargasso Sea [35, 43, 44, 53, 58].

The body condition of female silver eels was also not influenced by the second most prevalent parasite of our study, *A. clavula* (30 ± 7%), a native generalist acanthocephalan with *A. anguilla* as preferred definitive host [8, 11]. The apparent unchanged fitness of *A. clavula*-infected eels is in accordance with the absence of pronounced symptoms of disease generally observed for most fish infected by acanthocephalans (including those with high parasite intensities) [60 for review].

Component communities of metazoan parasites and body condition of silver eels widely varied between sites, suggesting differences in biocenosis (e.g., host species occurrence) and environmental constraints or stressors (including parasitism), with probable consequences on the spawning migration and reproduction success. Based on the inter-site differences and the virulence of *Pseudodactylogyrus* sp. and *A. crassus*, the silver eels from Stockholm Archipelago (Sweden) and from Burrishoole (Ireland) could be the most successful, whereas the migration success may be affected by the pathogenic effects of introduced parasites for the silver eels from the other sites studied, i.e., Frémur (France), Corrib (Ireland), and Gudenaa (Denmark). Indeed, only four out of the ten Swedish silver eels were infected and always with a very low parasite intensity. Moreover, they harbored no parasites on their gills and had both low *A. crassus* intensity (one helminth per swimbladder) and prevalence (three out of ten fish) suggesting low parasite pathogeny. Therefore, despite the low sample size, one can suppose that metazoan parasites have probably a limited influence on the condition of Swedish silver eels and on their migration success.

Burrishoole is considered as a pristine site with oligotrophic acid waters, no anthropogenic influence, and low levels of organic contaminants [46]. The growth rate of eels from this site is known to be extremely low [55], thus we consider that the one-year sooner sampling date (compared to the four other sites) had no significant impact on the eel parasite community and did not introduce a bias in our inter-site comparison. In our study, 90 ± 8% of the silver eels from Burrishoole were parasitized but this high total prevalence was not synonym of a strong parasitic constraint since they were mainly infected by the native intestinal acanthocephalan *A. clavula* (prevalence of 84 ± 10%, intensity of 31 ± 4) for which we demonstrated no influence on the host body condition. *A. crassus* was not recorded suggesting that the nematode may not complete its entire life cycle in Burrishoole despite its numerous intermediate crustacean (ostracods, copepods) and paratenic fish (and even snails) hosts [27, 49]. Moreover, no parasitic pathology probably occurred in the gills of Burrishoole silver eels because of the rarity of *Pseudodactylogyrus* sp. (only two infected out of the 49 specimens, harboring only one or three monogeneans).

Despite differences in the anthropogenic influence and the trophic status of Frémur (France), Corrib (Ireland), and Gudenaa (Denmark), *A. crassus* was omnipresent in *A. anguilla* from these sites with a prevalence varying from 89% (67/104) to 93% (80/99) probably inducing pathogenic effects [35 for review]. In addition, for 60% (36/81) and 71% (49/87) of the Corrib and Gudenaa silver eels, hypoxia due to the impaired gills was probably a major symptom as they were severely infected by *Pseudodactylogyrus* sp. with a mean intensity of 23 ± 12 and 19 ± 6 respectively. The parasite constraint appeared more substantial for the silver eels from Corrib and Gudenaa having both damaged swimbladder and gills than for those from Frémur since *Pseudodactylogyrus* sp. was absent.

Finally, based on the potential fitness loss induced by parasitism, we suppose that the migrant silver eels from Stockholm Archipelago (Sweden) and Burrishoole (Ireland) are able to contribute to the recruitment and gene pool of *A. anguilla* population, whereas those from our other study-sites [in particular from Corrib (Ireland) and Gudenaa (Denmark)] have a lower probability to reach the spawning grounds in Sargasso Sea.

Numerous aspects remain to be explored to explain the decline of the European eel population, probably induced by various interacting abiotic and biotic factors (e.g., habitat loss or fragmentation, changing hydrology, overfishing, pollution, and pathogens [9, 20, 21, 28, 36, 56]) and resulting in difficult and complex measures of preservation of this species. Enhancing the production of viable silver eels by water system represents the current target of the conservation strategy of the European eel [19]. However, we believe that the question of animal quality among river systems, which is presumed to influence the reproductive success, is also a key issue that must be urgently pursued for European eel conservation. Every potential impact on silver eel body condition warrants examination, more especially as synergistic effects can occur between environmental stressors such as parasites and contaminants, increasing the mortality of exposed organisms [47 for review].
